# Early Family Conflict and Behavioral Outcomes in Children from Low-Income Families: The Indirect Effects of Parental Depression and Parenting Practices

**DOI:** 10.3390/ijerph21121664

**Published:** 2024-12-13

**Authors:** Rong Huang, Rachel Chazan-Cohen, Delaina Carlson

**Affiliations:** 1 Department of Psychological Science and Counseling, Austin Peay State University, Clarksville, TN 37044, USA; 2 Department of Human Development and Family Sciences, University of Connecticut, Storrs, CT 06269, USA; rachel.c.cohen@uconn.edu (R.C.-C.); delaina.carlson@uconn.edu (D.C.)

**Keywords:** family conflict, internalizing, externalizing, detached parenting, intrusive parenting

## Abstract

Family conflict has been demonstrated as a risk factor impacting children’s mental health and behaviors; however, the mechanisms underlying these connections are unclear. Focusing on 1622 children from low-income families (51.4% boys, 38.3% White, 35.5% Hispanic/Latino, 22.1% African American, 4.1% other), the current study examines the role that maternal depression and parenting behaviors play in the associations between family conflict in early childhood and children’s internalizing and externalizing behaviors in early adolescence. Family conflict was positively associated with maternal depression at age 3 and detached parenting at age 5; however, maternal depression was linked to increased child internalizing and externalizing behaviors, and detached parenting was associated with decreased behavioral outcomes. Maternal depression at age 3 and intrusive parenting at age 5 successively mediated the association between family conflict and child externalizing. Multi-group analysis indicated different indirect paths of parenting behaviors in boys and girls. Specifically, in boys, the indirect effect of detached parenting on the links between family conflict and externalizing and internalizing behaviors was sustained. In girls, maternal depression and intrusive parenting sequentially explained the link between family conflict and externalizing behaviors. The findings highlighted the importance of addressing family well-being and parenting support, especially for children from low-income families.

## 1. Introduction

It is well documented that children with exposure to family conflict are at a greater risk for internalizing and externalizing behavioral problems [1,2,3,4,5,6]. Internalizing behavior is characterized by inwardly focused behaviors that are associated with emotional distress (i.e., anxiety, depression, social withdrawal, and/or somatic complaints; [7]), while externalizing behavior is a class of noncompliant outward behaviors (i.e., aggression, rule-breaking, and/or delinquent behavior; [8]). Most of the existing research is targeted at examining the concurrent or longitudinal relationship between family conflict and child behavioral outcomes, and less is known about how family conflict impacts children’s behavioral outcomes over time. It is important to investigate the underlying mechanisms of the long-term links between early family conflict and children’s behavioral outcomes. Family conflict is often associated with parental depression and negative parenting practices [9,10], both of which play important roles in promoting children’s negative behavioral outcomes [11,12]. There is a need for research exploring the indirect pathways of parental depression and negative parenting practices in the relationship between family conflict and child behavioral outcomes. The present study examined the longitudinal relations between family conflict in the first three years of life and children’s behavioral outcomes in fifth grade and the indirect effects of parent depression and negative parenting practices in an economically disadvantaged sample. We also further examined the invariance of these path models across genders to see if there are significant differences in terms of the pathways explaining the relationships between early family conflict and child behavioral outcomes. This study offers crucial insights into possible interventions for children who experienced family conflict in their early childhood years. 

### 1.1. Theoretical Framework

Economically disadvantaged families often experience higher levels of economic and psychological stressors, which can have significant negative impacts on children’s socio-emotional well-being [13]. The family stress model proposes that economic hardship negatively impacts children’s socio-emotional development through increased economic stress experienced by the family, which further impacts parents’ mental health and parenting behaviors [14,15]. Children in early childhood are particularly vulnerable to family environment, and poverty and its related risk factors may have lifelong impacts on children through family processes [16,17]. 

According to family systems theory, families are made up of individuals nested in interdependent systems [18]. Occurrences in one subsystem affect the other subsystems, such as the conflict between parents impacting the interactions within the parent–child dyads. This is partially explained through the spillover hypothesis, which suggests that mood, stress, and behaviors from one situation may be transmitted to another [19]. In the context of family conflict impacting child outcomes, spillover may happen directly when the negative emotions/behaviors from interparental conflict are transmitted to parent–child interaction and internalized by the children. Spillovers may also happen indirectly through factors that mediate the effect, such as parental mental health or parenting behaviors. Inspired by family systems theory, we proposed that family conflict in early childhood would directly and indirectly relate to children’s social behavioral outcomes over time. Derived from the family stress model, we are particularly interested in exploring the roles of maternal depression and parenting behaviors as mechanisms of the relationship between family conflict under age 3 and child behavioral outcomes in fifth grade in an economically disadvantaged sample.

### 1.2. Family Conflict and Child Behavioral Problems

Family conflict is often defined as disputes, tensions, criticism, or fights among family members. It is a broad concept that includes different types of infrafamilial relationships, such as marital conflicts, interparental conflicts, parent–child conflicts, as well as sibling conflicts [20,21]. Young children who are exposed to family conflict in early childhood may experience increased anxiety, insecurity, and unhealthy family relations and face an elevated risk of behavioral problems, such as aggression, in later childhood and adolescence [5,6,22,23,24,25]. Existing research on family conflict was often conducted on children from economically at-risk backgrounds, who are more likely to experience family conflict due to unstable financial status and limited resources at home. For instance, using the low-income sample from the Early Head Start Research Evaluation project sample, Streit et al. [6] demonstrated that family conflict experienced in the toddler years was linked to enhanced aggressive behaviors in fifth grade. Koblinsky et al. [24] found that a high level of family conflict has been associated with increased child externalizing problems in a sample of low-income African American preschoolers. Additionally, Formoso et al. [3] demonstrated that the positive relationship between family conflict and behavioral problems remained across diverse ethnic groups. 

Although the relationship between family conflict and internalizing behaviors has been less investigated, the existing research supported the close associations between family conflict and internalizing problems [2,26]. For instance, Tanaka and colleagues [26] demonstrated a concurrent positive link between family conflict and child anxiety in children aged 7 to 13 years old, and additionally, family conflict was associated with enhanced aggression, especially in children with a high level of anxiety. Formoso et al. [3] also found a positive connection between family conflict and child depression in adolescence. However, scarce research examined the long-term associations between family conflict in early childhood and child internalizing behaviors in adolescence. 

### 1.3. The Mediating Roles of Maternal Depression and Parenting Practices

In under-resourced living contexts, parents often face an increased risk of experiencing stress and depression, which can diminish the likelihood of effective, consistent, and supportive parenting [27,28]. Family conflict in low-income contexts may exacerbate these challenges and further compromise the quality of the family environment and parenting practices. Previous studies showed that family financial stress was often linked to maternal depression and negative parenting behaviors, such as detached or harsh parenting, which further leads to children’s externalizing behaviors [29]. Detached parenting emphasizes the emotional distance and lack of involvement and responsiveness, and intrusive parenting is characterized by excessive involvement and control in children’s thoughts and behaviors [30,31]. It is unclear if maternal depression and parenting practices explained the connections between family conflict and children’s behavioral adjustment. 

Both maternal depression and parenting behaviors are crucial factors in predicting child behaviors. Research showed that mothers with depressive symptoms were associated with more internalizing and externalizing problems in preschoolers [32,33,34,35,36,37]. For instance, Baker and colleagues [32] found a bidirectional relationship between maternal depression and child behavior problems across early childhood. In a systematic review, Goodman et al. [38] reviewed 37 studies that involved the links between a mother’s depression, parenting, and child functioning, supporting that maternal depression was closely and positively associated with a number of children’s behavioral outcomes, and negative parenting partially explained this relationship. Kuckertz and colleagues [36] also suggested that maternal depression often leads to increased psychological aggression in parenting, which in turn leads to more internalizing behaviors in children. To the contrary, low levels of maternal depression, high levels of maternal warmth, and supportive parenting have been linked with a reduction in internalizing problems and externalizing behavior [39,40,41]. For instance, in a sample of African American children in Head Start programs, Koblinsky and colleagues [24] found that lower levels of maternal depressive symptoms and more positive parenting were predictive of fewer externalizing and internalizing child behavior problems. 

A relatively small number of studies investigated the connections between family conflict and parents’ depression and parenting behaviors. Those studies found that family conflict was positively associated with parental depressive symptoms [42,43] and negative parenting [10,44]. In a daily diary study, Sears et al. [10] revealed that parents of 8 to 13 year olds were more likely to engage in negative parenting practices following an instance of family conflict. Additionally, Ayón et al. [44] used a person-centered approach to examine parenting profiles and Latino immigrant families’ family conflict. They found that more family conflict was associated with high levels of discipline and low engagement in parenting, while less family conflict was related to high levels of child-centered parenting. 

It is also likely that maternal depression and parenting may sequentially account for the long-term relationships between family conflict and child behavioral outcomes. Many studies have documented associations between maternal depression and higher levels of intrusiveness and detachment in parenting practices [45,46,47,48]. In a meta-analysis, Lovejoy et al. [49] found that mothers exhibiting symptoms of depression engaged in higher levels of negative and disengaged parenting behaviors as compared to mothers not experiencing depression. This is likely due to the significant negative impacts of depression on the parent’s cognitive, emotional, and behavioral functioning, which hinder their ability to provide sensitive, consistent, and supportive care [48]. However, no research so far examined how the long-term associations between family conflict in early childhood and both internalizing and externalizing behaviors in later childhood can be explained by maternal depression and negative parenting consecutively, leaving a significant gap in understanding the underlying processes through the long-term relationships between family conflict and children’s mental health.

### 1.4. Gender Differences

Abundant research supported that there are gender differences in how boys and girls respond to family stressors or family conflicts. In general, boys have been consistently found to exhibit higher levels of externalizing behaviors than girls, while girls show higher internalizing behaviors [50,51]. In a longitudinal study examining children from ages 6 to 10, Morelli and colleagues [5] demonstrated that the positive associations between family conflict and child externalizing behaviors were only found in boys, but not girls. However, family conflict was shown as a stronger predictor of children’s internalizing behaviors for girls than boys [21]. In another study that examined the relationship between maternal depression and child behavioral outcomes, Marchand and Hock [52] found a more consistent association between maternal depression and child internalizing behaviors across 4-year-old boys and girls, while maternal depression was only found to be positively associated with girls’ externalizing behaviors. A longitudinal study examining maternal depression and children’s externalizing behavior at age 2 and first grade found that the positive relationship between maternal depression and externalizing behavior was more pronounced in 2-year-old boys than girls. However, over time, this relationship in boys diminished, whereas this relationship in girls increased [53]. In a highly conflictual family, boys experiencing maternal depression were more likely to show increased externalizing behaviors by kindergarten, whereas for girls, exposure to maternal depression was more indicative of later internalizing behaviors [54]. 

Although much research has examined the gender difference in child behavioral outcomes due to challenging family contexts [5,50,55], few studies have investigated whether the associations between family conflict and children’s behavioral outcomes operate through different pathways in boys and girls. In terms of parenting, research supported that parents tend to use more assertive discipline and be more controlling of boys than of girls [56,57]. Gender differences also exist in the relationships between maternal factors and child behavioral outcomes. For instance, differential parenting strategies in boys and girls often lead to different effects on children’s behavioral outcomes [58]. Specifically, higher levels of negative parenting and lower levels of warmth were more likely to be associated with increased externalizing behaviors among boys than girls [58,59]. Another study of older children samples (aged 9 to 15 years) conducted by Gruhn and colleagues [60] demonstrated that maternal depression was positively related to mothers’ detached parenting for both boys and girls, while maternal depression was more likely to be related to enhanced intrusive parenting for parents of boys. Given these identified gender differences, it is reasonable to expect that the pathways of family conflict predicting children’s behavioral outcomes differ in boys and girls. 

### 1.5. The Current Study 

The current study contributes to expanding previous studies in several ways. First, this is a longitudinal design spanning from toddlerhood to adolescence, and few studies have examined the long-term associations between family conflict and child behavioral outcomes over such an extended period. Second, no studies, to our knowledge, have explored maternal depression and negative parenting practices as serial mediators in the longitudinal relationships between family conflict and child behavioral outcomes. In addition, previous research has demonstrated gender differences in parenting practices and behavioral problems [51,56,61], while no studies have explored the gender differences in the relationships and pathways between family conflict and child internalizing and externalizing behaviors. 

To address gaps in the literature and expand our understanding of the underlying pathways between family conflict and child behavioral adjustment in a low-income sample, we used an ethic-racially diverse sample from the Early Head Start Research Evaluation Program and proposed three research questions as follows:

(1) What are the associations between family conflict across toddlerhood and children’s internalizing and externalizing outcomes in fifth grade? We hypothesized that early family conflict would be positively associated with children’s internalizing and externalizing behaviors reported in early adolescence.
(2) Do maternal depression and negative parenting practices account for this long-term relationship consecutively? We anticipated that high levels of family conflict in early childhood would be associated with increased maternal depression when children were at age 3 and increased negative parenting practices when children were at age 5 successively, which in turn relates to increased behavioral problems in early adolescence. More specifically, based on existing literature, we expected that intrusive parenting would be positively associated with children’s externalizing behaviors, whereas detached parenting would be positively linked to children’s internalizing behaviors.
(3) Do the mediating roles of maternal depression and negative parenting in the relationship between family conflict and child behavioral outcomes differ in boys and girls? Based on the existing literature, we expected that there would be gender differences in the mediation model. Specifically, among boys, the links between family conflict and child behavioral outcomes will be positively explained by detached parenting and maternal depression, while for girls, the relationships between family conflict and child behavioral outcomes are likely to be explained by enhanced intrusive parenting and maternal depression.

**Figure 1:**
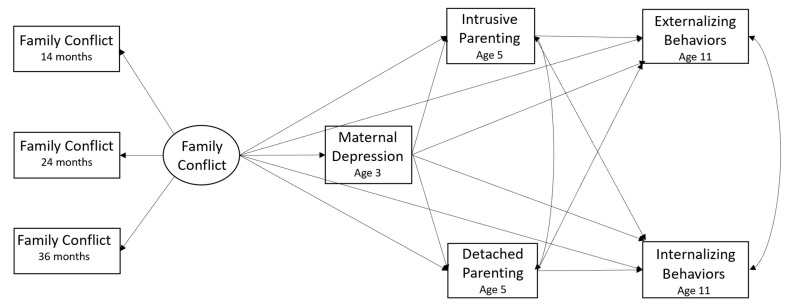
The conceptual model for the direct and indirect relations between early childhood family conflict and children’s behavioral outcomes at age 11.

Figure 1 presents the conceptual model. The latent variable of family conflict was measured by three indicators: family conflict when children were at age 14, 24, and 36 months. For all the paths, we controlled for the Early Head Start (EHS) program, program site, child gender, child age, and race/ethnicity. 

## 2. Materials and Methods

### 2.1. Participants

A total of 3001 low-income families participated in the Early Head Start Research and Evaluation Project (EHSREP), which is a longitudinal evaluation study of the Early Head Start programs from 17 sites across the United States [62]. The use of secondary data for this study was approved by the Institutional Review Board (IRB Protocol Number: X22-0195) at the University of Connecticut. We excluded 1379 cases due to (1) the primary applicants for the study were not mothers, such as grandparents or fathers (*n* = 17), (2) had no data on grade 5 and at least one other earlier wave (*n* = 1362). The analytical sample of this study consists of 1622 children (51.4% males). Among them, 832 children (51.3%) were randomly assigned to the EHS program, and the rest (49.7%) were assigned as the control group. The demographic information of the sample is presented in Table 1.

### 2.2. Measures

#### 2.2.1. Family Conflict in the Early Three Years

Caregivers reported on family conflict within the household using the Family Environment Scale (FES; [63]) when children were 14, 24, and 36 months. The Family Conflict subscale of the Family Environment Scale included 5 items on a 4-point scale, asking the extent to which the public expressions of negative emotions and behaviors, such as anger or conflictual interactions in the household. The response 4 indicates higher levels of agreement with statements such as, “We fight a lot”, and “We hardly ever lose our tempers”. The internal consistency reliability for the family conflict in the three waves ranged from 0.67–0.68. Responses in each wave were coded and averaged so that a higher score indicates a high level of conflict [64]. 

#### 2.2.2. Maternal Depression at Age 3

Maternal depression was assessed by the Center for Epidemiological Studies Depression Scale—Short Form (CES-D-SF; [65,66]), which was completed by mothers when their children were at age 3. The scale contains 12 items, asking the respondents how often they had a particular symptom of depression, such as loneliness, sadness, restless sleep, poor appetite, and lack of energy, in the past week. Each item was accompanied by a 4-point scale (0 = rarely, 1 = some, 2 = occasionally, 3 = most). The internal consistency for the current sample was 0.88. Total score ranged from 0 to 36, and the higher scores indicate a greater number of depressive symptoms.

#### 2.2.3. Parenting Practices at Age 5 

Parenting behaviors in a 10 min videotaped parent–child play activity (the play doh task) were observed and rated using an observational rating [67]. We used two main subscales of parenting behaviors: *intrusiveness* and *detachment.* The parental intrusiveness subscale measures the degree to which the parent controls the children’s actions during play. The scores ranged from 1 to 7, with higher scores indicating that the parent is more intrusive during the play and the child shows no self-direction. The parental detachment subscale measures the parents’ awareness, attention, and/or engagement with the child during the play. The scores ranged from 1 to 7, with higher scores indicating that the parent almost pays little attention to the child or does not engage in the play with the child. 

#### 2.2.4. Behavioral Problems at Age 11 

When participants reached fifth grade, parents reported on children’s internalizing and externalizing behaviors using the Child Behavior Checklist [7]. Externalizing behavior is a composite score for subscales of aggression and rule-breaking behaviors. Internalizing behavior is a composite score for the subscales of anxious/depressed, withdrawn/depressed, and somatic complaints. The internal consistency for externalizing (alpha = 0.91) and internalizing subscales (alpha = 0.85) was excellent. 

### 2.3. Analytical Plan

The EHSREP data and detailed information on all the measures are publicly available at the Henry A. Murray Research Archive and Child and Family Data Archive: https://www.childandfamilydataarchive.org/cfda/archives/cfda/studies/3804/summary (accessed on 20 September 2022). All the analysis was conducted in R Studio [68]. In the analytical sample (N = 1622), all participants participated in each wave of data collection, with varying levels of missing data in different variables. The rates of missing data for child externalizing and internalizing behaviors were quite low (0.6%), while the parent-related variables and family conflict at the first three time points were relatively high (i.e., ranging from 14.8% to 25.8%). The missingness was not related to any variables of interest. Little’s MCAR test indicated that the pattern of missingness was completely random, *p* = 0.11. To address the missingness, multiple imputation (MI) with 20 imputations was conducted.

To address the first two research questions, we conducted a structural equation modeling (SEM) to investigate if early childhood family conflicts have long-term associations with children’s behavioral problems and whether maternal depression at age 3 and maternal parenting practices at age 5 have indirect effects on the long-term relationships successively. To explore the gender differences in the proposed model, we conducted a multigroup SEM to see how the model paths differed in boys and girls. A chi-square test was conducted to examine if there were significant differences between the baseline model with none of the parameters constrained to be equal across groups and the constrained model that specified all the parameters to be constrained across groups. A significant χ^2^ difference between the less restrictive and more restrictive models indicates that the null hypothesis that the parameters are equal across boys and girls is rejected. We used a range of model fit indices, including the comparative fit index (CFI, >=0.90), the root mean square error of approximation (RMSEA; <0.06), and the standardized root mean squared residual (SRMR; <0.08) to determine if the model fit was good enough [69]. The indirect effects were examined through the 95% confidence interval, using bootstrapping with 5000 samples: A 95% CI not including 0 indicates a significant mediation pathway. 

## 3. Results

### 3.1. Descriptive Analysis and Correlations

Descriptive information and correlations among the key variables are presented in Table 2. Family conflicts and parent depression at age 3 were normally distributed based on the criteria that skewness and kurtosis within −2 and 2 [70]; however, intrusive parenting, detached parenting, and child behavior outcomes are highly peaked (i.e., kurtosis is larger than 2). This pattern has been found in this dataset and others when looking at these uncommon parenting behaviors [71]. Ratings of family conflict in the first three years were moderately related to each other (*ps* < 0.001). Family conflicts were correlated with maternal depression at age 3 (*ps* < 0.001), parent detachment at age 3 (*ps* < 0.05), child internalizing behaviors (*ps* < 0.001), and externalizing behaviors at age 11 (*ps* < 0.001). Small-sized positive correlations were found between parent intrusiveness and family conflict at age 2, *r* = 0.11, *p* < 0.001, and between parent intrusiveness and family conflict at age 3, *r* = 0.064, *p* = 0.010. Maternal depression was positively related to parent intrusiveness, with a small effect size, *r* = 0.10, *p* < 0.001, but not related to detachment in parent–child interactions, *r* = 0.03, *p* = 0.237. In terms of the correlations with child outcomes, maternal depression was moderately and positively associated with both child internalizing and externalizing behaviors (*ps* < 0.001). Interestingly, detached parenting at age 5 has a small negative association with child internalizing behaviors at age 11, *r* = − 0.078, *p* = 0.002, but not child externalizing behavior, *r* = −0.039, *p* = 0.118, whereas parent intrusiveness has a slight positive association with externalizing behaviors at age 11, *r* = 0.107, *p* < 0.001, but not internalizing behaviors at age 11, *r* = −0.015, *p* = 0.548. 

### 3.2. Path Analysis

#### 3.2.1. Direct Paths

**Figure 2:**
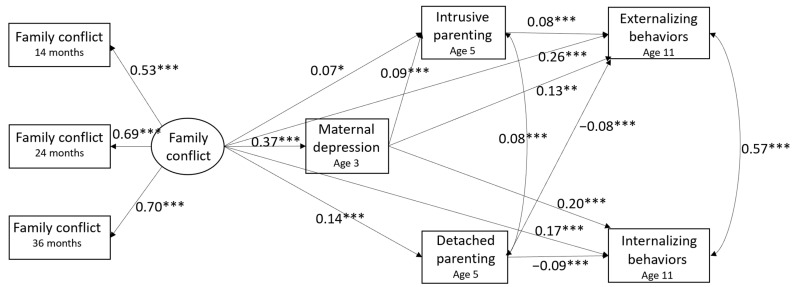
Direct and indirect paths between early family conflict and children’s internalizing and externalizing behaviors. Standardized coefficients are reported in the model. The model controlled for EHS program, program site, child gender, child age, and race/ethnicity. * *p* < 0.05, ** *p* < 0.01, *** *p* < 0.001.

The structural equation model indicated that the hypothesized model fit the data well, χ^2^(73) = 240.14, *p* < 0.001, CFI = 0.932, RMSEA = 0.038, SRMR = 0.020. Figure 2 summarizes the model paths and standardized coefficients. All the paths were controlled for the EHS program, program site, child gender, child age, and child race/ethnicity. Results showed that early family conflict had positive direct associations with maternal depression at age 3, intrusive and detached parenting at age 5, and both child internalizing and externalizing behaviors at age 11. Maternal depression was positively related to intrusive parenting but not detached parenting. Additionally, maternal depression was associated with both child internalizing and externalizing behaviors. There was a direct positive association between intrusive parenting and externalizing behaviors, while the link between intrusive parenting and internalizing behavior was not significant. Detached parenting was negatively associated with both internalizing and externalizing behaviors.

#### 3.2.2. Indirect Paths

The coefficients and bootstrapping confidence intervals for the indirect paths were obtained (see Table 3) to examine whether maternal depression and negative parenting practices mediated the relationships between early family conflict and child behavioral outcomes. Results showed that maternal depression was a strong mediator for both the associations between family conflict and child internalizing behaviors, 95% CI [0.283, 0.571], and the association between family conflict and child externalizing behaviors, 95% CI [0.226, 0.557]. Detached parenting was also a significant mediator for the association between family conflict and internalizing behavior, 95% CI [−0.142, −0.025], and the association between family conflict and externalizing behavior, 95% CI [−0.198, −0.027], although the direction indicates that more detached parenting was associated with less internalizing and externalizing behaviors. However, intrusive parenting only mediated the association between family conflict and externalizing behaviors, 95% CI [0.002, 0.095], but not the link between family conflict and internalizing behavior, 95% CI [−0.038, 0.010]. Regarding the double-mediator pathways, we found that the relationship between family conflict and externalizing behavior was partially explained through maternal depress ion and intrusive parenting successively, 95% CI [0.004, 0.042]. 

### 3.3. Multigroup Analysis

**Figure 3:**
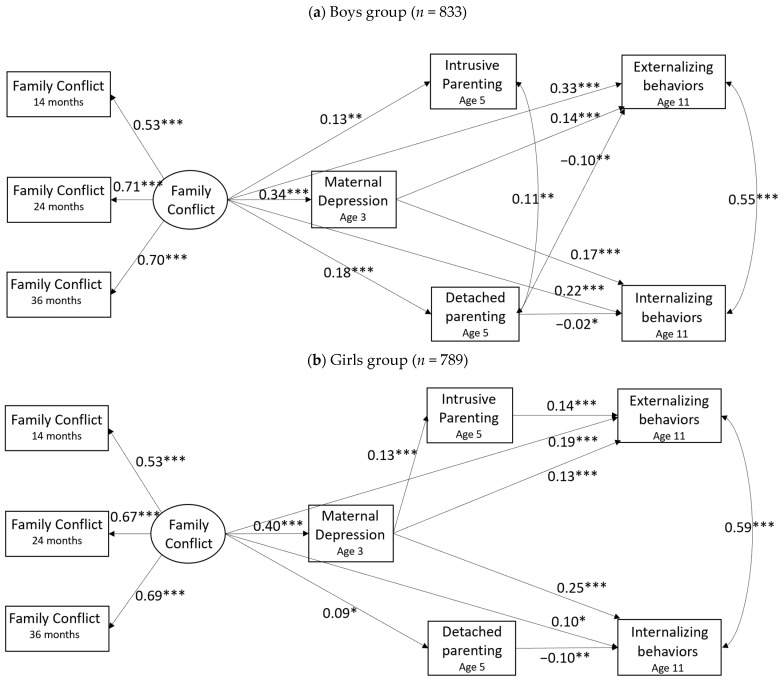
Comparisons of the model path coefficients in boys (**a**) and girls (**b**) groups. Standardized coefficients are reported in the models. Both models controlled for EHS program, program site, and child race/ethnicity. * *p* < 0.05, ** *p* < 0.01, *** *p* < 0.001.

Multigroup analysis examining whether there were differences across boys and girls between the unconstrained model (χ^2^(140) = 324.32, CFI = 0.924, RMSEA = 0.040, SRMR = 0.022) and the constrained model (χ^2^(260) = 479.88, CFI = 0.91, RMSEA = 0.032, SRMR = 0.027) indicated that the model paths differed by child gender, Δχ^2^(120) = 155.56, *p* = 0.016. Figure 3a,b summarizes the standardized coefficients of paths for the boys and girls groups. The model fit both boys and girls data well (boys: χ^2^(70) = 142.54, *p* < 0.001, CFI = 0.932, RMSEA = 0.035, SRMR = 0.021; girls: χ^2^(70) = 181.72, *p* < 0.001, CFI = 0.906, RMSEA = 0.045, SRMR = 0.024). For both boys and girls, there were direct associations between family conflict and children’s internalizing and externalizing outcomes. Additionally, maternal depression remained a strong mediator that accounted for these relations across child gender. 

The indirect paths through parenting practices differed across boys and girls. Specifically, among boys, although intrusive parenting was associated with family conflict, it was not a significant mediator in the relationships between family conflict and child externalizing behavior, 95% CI [−0.051, 0.103], or internalizing behaviors, 95% CI [−0.076, 0.033]. Instead, detached parenting indirectly explained the associations between family conflict and both internalizing, 95%CI [−0.337, −0.034], and externalizing behaviors, 95% CI [−0.197, −0.013]. For girls, the association between family conflict and child externalizing behaviors was partially mediated by maternal depression and intrusive parenting successively, 95% CI [0.014, 0.115], while detached parenting was no longer a strong mediator explaining the relationships between family conflict and either externalizing, 95% CI [−0.138, 0.012] or internalizing behaviors, 95% CI [−0.136, 0.005].

## 4. Discussion

Although it is widely accepted that family conflict has concurrent and long-term impacts on children’s behavioral outcomes, less is known about the mechanisms underlying these associations. This current study, using a subsample from the longitudinal EHSREP dataset, examined maternal depression and parenting practices as possible mediators for the associations between early childhood family conflict and internalizing and externalizing behavior outcomes in early adolescence. Partially consistent with our hypothesis, the data provide evidence that for the full sample from low-income families, both maternal depression and parenting practices indirectly explained the associations between early family conflict and children’s internalizing and externalizing outcomes, though unexpectedly detached parenting led to fewer parent-reported behavioral problems. Moreover, the nuanced indirect effects differed across child gender. As expected, detached parenting played an important mediating role in the relationship between family conflict and behavioral problems among boys, while in girls, the relationship between family conflict and externalizing behavior was explained by maternal depression and intrusive parenting sequentially.

With the strength of this longitudinal study, we were able to find that family conflict in early childhood strongly predicts both internalizing and externalizing behaviors later in early adolescence, for both boys and girls. This adds to existing evidence that a negative family climate in early childhood poses an increased risk for children’s behavioral problems, even over a long period. A number of studies have demonstrated that family conflict is associated with children’s aggression or externalizing behaviors over time [6,22], while our study highlighted that the long-term relationship was also found between family conflict and child internalizing behavior. More importantly, these longitudinal relationships remained regardless of child gender, which contradicts the findings from Morelli et al. [5], who focused on elementary children and only found the relationship between family conflict and externalizing behaviors in boys. This may be due to the characteristics of our samples. Morelli et al.’s [5] sample was at higher risk of child maltreatment and violence exposure. This again underscores the notion that the family climate in the early years is critical in children’s later development. It also calls for a better understanding of the mechanisms of these robust longitudinal relationships between early family conflict and children’s behavioral outcomes and across genders. 

One of our most notable findings was that overall, the indirect paths of maternal depression and parenting practices in these longitudinal relationships between family conflict and child behavioral outcomes. Family conflict in early childhood was positively related to maternal depression when children were at the age of 3, which further contributes to children’s behavioral outcomes in early adolescence, including both internalizing and externalizing behaviors. These results are in accordance with previous research that family conflict was associated with elevated maternal depressive symptoms [42,43] and that maternal depression is an important predictor of children’s internalizing and externalizing behaviors [33,36,37,38].

Additionally, family conflict in early childhood was connected to a higher chance of intrusive parenting in mothers, which was associated with increased child externalizing behavior but not internalizing behavior. Moreover, the long-term association between family conflict and child externalizing behavior can also operate through the paths of maternal depression and intrusive parenting consecutively over time. These findings highlight the unique role of intrusive parenting in explaining the associations between family conflict and children’s externalizing behaviors. Previous research has demonstrated the strong association between intrusive parenting and externalizing behaviors in children [31,60]. In the context of family conflict, mothers with intrusive parenting are modeling an ineffective way, in the form of overt controlling behaviors, to deal with stress and emotions, which increases children’s externalizing behaviors, such as aggression or defiance, rather than internalizing their negative emotions. Jiang and colleagues [72] conducted a meta-analysis based on 55 studies focusing on early childhood and found that mothers’ intrusive parenting was associated with children’s externalizing behaviors but not children’s internalizing behaviors. This suggests that children are more likely to take their mother’s intrusive parenting overtly and model observable behaviors (e.g., aggression) rather than covertly, leading to anxiety or depression. 

Interestingly, in our low-income overall sample, although mothers’ detached parenting was also a strong mediator for the association between family conflict and both behavioral outcomes, the directions were negative. That is, detached parenting in mothers, associated with high family conflict, predicted fewer internalizing and externalizing behaviors in children. This counterintuitive finding may be due to several reasons. Detachment in mothers might inadvertently reduce the intensity of conflicts, giving them space to navigate their emotions and stress and avoiding transmitting negative feelings/emotions to children. Additionally, children living in a negative and conflict-ridden family climate who experience maternal detachment might develop resilience that helps them manage emotions and regulate behaviors independently. It is also possible that children seek some external support from extended family or school systems, which helps them to focus on the positive aspects of their environment and maintain a positive relationship with others; it mitigates the possible negative impacts of the home environments with conflicts. One other explanation for this finding is that the child behavioral outcomes were all based on parent reports. For those parents who were detached from their child, they may not have a close relationship with the child, which may lead to inaccurate reports of children’s internalizing and externalizing behaviors. 

Our study further demonstrated that different mechanisms, especially maternal parenting practices, explained the long-term associations between early family conflict and later child behavioral outcomes for girls and boys. Particularly, in boys, maternal depression and detached parenting are critical independent mediators in the relationship between family conflict and both behavioral problems. While in girls, we only found a consecutive indirect effect through maternal depression and intrusive parenting on the association between family conflict and externalizing behavior. It suggests that experiencing a family conflict context in early childhood; boys’ and girls’ behavioral outcomes may differentially relate to different parenting practices. Maternal detachment in a household with high levels of family conflict tends to be associated with boys’ behavioral regulation and adjustment. This is consistent with the overall sample findings, suggesting that the indirect effect of detached parenting may be driven by the boys group. Although family conflict was also linked to intrusive parenting in mothers, which did not significantly relate to either internalizing or externalizing behaviors in boys. This is inconsistent with our expectations or previous research on parenting and child behavioral outcomes [58]. It is likely that other important factors that we did not examine, for instance, fathers’ intrusive parenting, have overridden these effects. It is also likely that boys tend to cope with family conflict and the related intrusive parenting rationally; for instance, they may tend to internalize their distress or express it in appropriate ways, such as doing sports.

The overall model showed that in the context of high levels of family conflict, mothers with depressive symptoms tend to practice intrusive parenting while interacting with their children, which was associated with enhanced externalizing behaviors in children. The gender-comparison models suggest that this effect may be driven by girls. It is worth noting that family conflict was not directly associated with mothers’ intrusive parenting in girls but through maternal depression. It highlights the unique combination role of maternal depression and intrusive parenting in contributing to girls’ externalizing behavior. Girls are often socialized to be emotionally sensitive to interpersonal relationships [73]; they may feel more emotional distress than boys when facing family conflict, maternal depression, and/or intrusive parenting. Consequently, girls need more support to cope with these challenges and stress at home, other than modeling mothers’ intrusive behaviors and reacting aggressively to express their stress/distress. 

### 4.1. Implications

This study has important implications for parental support programs and prevention/intervention research, particularly in economically disadvantaged families who are at increased risk of experiencing higher rates of family conflict. First, the findings highlight the robust mediating role of parent depression and parenting practices in the associations between early family conflict and the child’s later behavioral outcomes for the overall sample. This suggests a pressing need to prioritize parents’ mental health and positive parenting in these environments. For instance, by providing sufficient mental health support and targeted parenting training, interventions can help foster healthier family dynamics and promote children’s positive development, even in low-income households where family conflict is prevalent. Letting parents know about the detrimental effects of early family conflict on children’s development as well as the importance of having a supportive connection with the child is essential. Then, giving parents specific skills and strategies for managing conflict and engaging in positive interactions with their children promotes positive outcomes for children. Even in the context of elevated family conflict, supportive parenting can buffer the negative effects of family conflict on children’s internalizing and externalizing behaviors. 

The study also underscores gender differences in how children cope with family conflicts and related parenting practices, which indicates that boys and girls may require different approaches to support their behavioral adjustment in the context of family conflict. The findings imply that for girls, it may be beneficial to practice less over-controlling and intrusive parenting and to build stronger, supportive, and nurturing relationships with the parents. Whereas boys may likely benefit from appropriate space and emotional distance from mothers during periods of conflict. Given the unexpected findings on detached parenting in boys, more research would be needed to validate these observations. Overall, these insights are crucial for directing future research and developing tailored interventions that address the unique emotional and behavioral needs of boys and girls in the context of family conflict. 

### 4.2. Limitations and Future Directions 

Although this study suggests important practical implications, several limitations should be noted. First, except for parenting practices, other constructs (i.e., family conflict, maternal depression, and children’s behavioral outcomes) were all measured based on parent reports. To validate and build upon these findings, future researchers should utilize diverse methodologies to evaluate family conflict (such as observation) and child behavior problems (such as teacher reports and child reports). Employing various assessment approaches will provide a more thorough understanding of these constructs and their interactions, thereby improving the overall reliability and validity of the research [74]. Second, we only considered the mechanisms of mothers’ depression and their parenting practices, which inevitably may not provide a complete insight into the explaining factors in the relationship between family conflict and child behavioral adjustment. Future studies may consider incorporating both mothers’ and fathers’ parenting and their interactions with children and see how their parenting practices contribute to children’s externalizing and internalizing behaviors and across genders. To enhance the depth of analysis and compare the differences in the sizes of indirect paths across gender, future investigation using moderated mediation is encouraged [75]. 

Additionally, although this secondary data collected information over time, we are not able to demonstrate the causal effects between family conflict and child behavioral outcomes, as well as the causal paths through parental depression and parenting practices. It is highly possible that family conflict in early childhood could be related to later child outcomes because the family conflict is stable over time. The dataset also restricts us to examining the bidirectionality of family conflict and children’s behavioral problems over time, but we cannot refute the possibility that children’s early temperament and behavior contribute to maternal depression, intrusive parenting, and family conflict among family members, which further leads to child behavioral problems later on. Future studies may consider using more appropriate methodology to explore the causation effect and including intervention and prevention programs focusing on positive family climate and caregiver mental health for families with high levels of conflict. It would be helpful to identify effective ways to promote family well-being and child well-being.

## 5. Conclusions

Overall, the findings of the current study contribute to the current literature by exploring the mechanisms of maternal depression and parenting practices in the longitudinal associations between family conflict assessed in early childhood and children’s internalizing and externalizing behaviors in later school years. Further, this study provided support that boys and girls in the context of family conflict are prone to being impacted by different parenting practices, which contributes to varied social-emotional behaviors. These findings underscore the importance of providing a positive and healthy family climate in children’s early years. Moreover, it offers educational implications on how to better promote children’s social-emotional outcomes for those experiencing high levels of family conflict. 

## Figures and Tables

**Table 1:** Program, child, and family characteristics (N = 1622).

Characteristics	Percentage/M (SD)
EHS program (yes)	51.3%
Child age (months) at random assignment	3.39 (4.72)
Child male	51.4%
Race/ethnicity	
White	38.3%
Hispanic/Latino	35.5%
African American	22.1%
Other	4.1%
Mother’s age (years) at random assignment	22.7 (5.62)
Teenage mom (yes)	37.8%
Education level	
Less than high school	43.2%
High school or GED	31.2%
More than high school	25.6%
Employment status	
Employed	25.3%
In school/training	24.2%
Not employed	50.5%
Household income at Pre-K	$27,807 ($20,129.3)
Living status at Pre-K	
Living alone	28.5%
Living with spouse or partner	71.5%

Note: GED = general educational development; Pre-K = pre-kindergarten.

**Table 2:** Descriptives and correlations among key variables.

	M (SD)	1	2	3	4	5	6	7
Family conflict—age 1	1.72 (0.49)							
Family conflict—age 2	1.71 (0.49)	0.389 ***						
Family conflict—age 3	1.67 (0.49)	0.338 ***	0.487 ***					
Maternal depression— age 3	7.72 (6.65)	0.233 ***	0.209 ***	0.297 ***				
Parent intrusiveness— age 5	1.77 (0.80)	0.007	0.106 ***	0.064 *	0.097 ***			
Parent detachment—age 5	1.36 (0.69)	0.054 *	0.073 **	0.101 ***	0.029	0.114 ***		
Child internalizing behaviors— age 11	5.79 (5.11)	0.201 ***	0.118 ***	0.156 ***	0.269 ***	−0.015	−0.078 **	
Child externalizing behaviors— age 11	8.01 (7.95)	0.213 ***	0.196 ***	0.234 ***	0.263 ***	0.107 ***	−0.039	0.612 ***

Note: * *p* < 0.05, ** *p* < 0.01, *** *p* < 0.001.

**Table 3:** Indirect effects of family conflict on children’s behavioral outcomes.

	Coeff.	SE	95% CI
Family conflict → age 3 maternal depression → age 11 externalizing	0.383	0.085	[0.226, 0.557]
Family conflict → age 5 intrusive parenting → age 11 externalizing	0.043	0.024	[0.002, 0.095]
Family conflict → age 5 detached parenting → age 11 externalizing	−0.093	0.044	[–0.198, –0.027]
Family conflict → age 3 maternal depression → age 11 internalizing	0.419	0.073	[0.283, 0.571]
Family conflict → age 5 intrusive parenting → age 11 internalizing	−0.008	0.012	[−0.038, 0.010]
Family conflict → age 5 detached parenting → age 11 internalizing	−0.072	0.030	[–0.142, −0.025]
Family conflict → age 3 maternal depression → age 5 intrusive parenting → age 11 externalizing	0.020	0.009	[0.004, 0.042]
Family conflict → age 3 maternal depression → age 5 detached parenting → age 11 externalizing	0.006	0.008	[−0.008, 0.026]
Family conflict → age 3 maternal depression → age 5 intrusive parenting → age 11 internalizing	−0.003	0.005	[−0.014, 0.005]
Family conflict → age 3 maternal depression → age 5 detached parenting → age 11 internalizing	0.005	0.006	[−0.007, 0.019]

Note: Coeff. = unstandardized coefficient; SE = standard error; CI = confidence interval. The significant mediation pathways are indicated by a 95% confidence interval that does not include 0.

## Data Availability

The EHSREP data and detailed information on all the measures is publicly available at the Henry A. Murray Research Archive and Child and Family Data Archive: https://www.childandfamilydataarchive.org/cfda/archives/cfda/studies/3804/summary (accessed on 20 September 2022).
